# CID-GCN: An Effective Graph Convolutional Networks for Chemical-Induced Disease Relation Extraction

**DOI:** 10.3389/fgene.2021.624307

**Published:** 2021-02-10

**Authors:** Daojian Zeng, Chao Zhao, Zhe Quan

**Affiliations:** ^1^Hunan Provincial Key Laboratory of Intelligent Computing and Language Information Processing, Hunan Normal University, Changsha, China; ^2^School of Computer and Communication Engineering, Changsha University of Science and Technology, Changsha, China; ^3^College of Information Science and Engineering, Hunan University, Changsha, China

**Keywords:** relation extraction, graph convolutional network, chemical-induced disease, inter-sentential relation, document level

## Abstract

Automatic extraction of chemical-induced disease (CID) relation from unstructured text is of essential importance for disease treatment and drug development. In this task, some relational facts can only be inferred from the document rather than single sentence. Recently, researchers investigate graph-based approaches to extract relations across sentences. It iteratively combines the information from neighbor nodes to model the interactions in entity mentions that exist in different sentences. Despite their success, one severe limitation of the graph-based approaches is the over-smoothing problem, which decreases the model distinguishing ability. In this paper, we propose CID-GCN, an effective Graph Convolutional Networks (GCNs) with gating mechanism, for CID relation extraction. Specifically, we construct a heterogeneous graph which contains mention, sentence and entity nodes. Then, the graph convolution operation is employed to aggregate interactive information on the constructed graph. Particularly, we combine gating mechanism with the graph convolution operation to address the over-smoothing problem. The experimental results demonstrate that our approach significantly outperforms the baselines.

## 1. Introduction

Chemical-disease relation (CDR) plays an essential role in various areas of biomedical research and health care (Dogan et al., 2009). Understanding correlations between chemicals and diseases is made challenging. At present, it provides manually curated facts about CDR in the commonly used bioinformatics databases such as the Comparative Toxicogenomics Database (CTD) (Davis et al., 2017). Nevertheless, with the rapid accumulation of the biomedical literature, the manual curation not only time consuming, but also requires professional labeling staff and insufficient to keep up-to-date. Automatic extraction of CDR has attracted plenty of attention and become increasingly important.

To promote the research,the BioCreative V in 2015 proposed a new challenge for extracting CDR from the biomedical literature. The challenge included two subtasks (Wei et al., 2015): the disease named entity recognition (DNER) task and the chemical-induced disease (CID) relation extraction task. The former one is to identify diseases and chemicals from the given raw PubMed abstracts and normalize them to Medical Subject Headings (MeSH) concept identifiers. The latter one is to assess whether there is association between chemicals and diseases denoted by MeSH identifier pairs. In this paper, we mainly focus on the CID relation extraction task.

The CID relation extraction is usually formulated as a binary classification problem. The difficulty of the task is to get a good vector representation for a pair of chemical and disease. Different from previous biomedical relation extraction tasks such as protein-protein interaction (PPI) detection and drug-drug interaction(DDI) detection, the CID relations are determined at document level, i.e., an entity is often represented in multiple mentions and the relations could be described across sentences. The main challenge of document level relation extraction is to deal with multiple entity mention pairs of the same concepts all over a document and capture inter-sentence relations when two entities are not in the same sentence.

Traditional methods handle CID relation extraction as two separated tasks (intra- and inter-sentence relation extraction) (Zhou et al., 2015; Qian and Zhou, 2016; Gu et al., 2017). The results of these two subtasks are merged through a post-processing way to obtain CID relations between entity concepts at document level. The development of such methods mainly draws on the traditional sentence level relation extraction. Feature-based methods (Qian and Zhou, 2016) and kernel-based methods (Zhou et al., 2015) have appeared one after another. With the revival of deep learning in recent years, researchers have used various neural networks, such as convolutional neural networks (Gu et al., 2017), to automatically learn features. The separated framework classifies multiple mention-level pairs, which is simple and easy to implement. However, it ignores the interactions in multiple mentions of the target entities in different sentences, which are especially useful to identify the inter-sentential relations.

In order to take full advantage of correlations among different mentions in a document, the graph-based approaches are proposed for CID relation extraction. The graph-based models interpret words as nodes and edges as intra- and inter-sentential relations between the words. Quirk and Poon (2017) build a document graph with different dependency edges. Following this work, researchers exploit graph LSTM (Peng et al., 2017), graph state LSTM (Song et al., 2018) or RNNs on dependency tree structures (Gupta et al., 2019). These methods can simultaneously capture intra- and inter-sentential features, but they do not aggregate the features of multiple mentions. Recently, many approaches are proposed to address this problem. Christopoulou et al. (2019a) is one of the most powerful systems, which use an edge-oriented graph (EoG) neural model to learn the representation of mention pairs.

Although edge-oriented models such as *EoG* have good performance, it only focuses on the edge representation of the graph and ignores the representation of the nodes in the graph. On the one hand, with the multi-hop reasoning over the document graph, the meaning of some edges on the path of multiple entity pairs will become overlapping and vague. On the other hand, since it only focuses on the representation of the node pair, it may lose the specific and related information (e.g., entity or mention type) of the node itself, which is very important in document-level relation extraction. For learning the representation of the nodes in the graph, a powerful approach is Graph Convolutional Networks (GCNs), which have achieved state-of-the-art results in various application areas on real-world datasets. The basic idea is to carry out convolution filtering on the graph and update the node representations by propagating information between nodes. Besides, by treating objects as nodes and connecting related nodes, GCNs can be adopted to various graph-based multi-hop inference tasks. However, most of the state-of-the-art GCN models are shallow due to the over-smoothing problem. The over-smoothing means that after multi-layer graph convolution, the effect of Laplacian smoothing causes node representation toward a space that contains limited distinguished information. This issue also drives the GCN difficult to model the relation between long-distance nodes, which is critical in CID relation extraction.

In this paper, we propose an effective Graph Convolutional Networks (GCNs) for CID relation extraction (CID-GCN). Similar to Christopoulou et al. (2019a), we construct a heterogeneous graph which contains mention, sentence and entity nodes. By processing all the entities and mention nodes in the document in a unified manner, the intra- and inter-sentence relation facts can be extracted simultaneously in a model. Instead of using a walk-based method, we subsequently exploit graph convolution operation to aggregate interactive information on the constructed graph. Graph convolution operation applies the same linear transformation to all the neighbors of a node followed by a non-linear activation function. In order to make the graph better adapt to CID relation extraction, we stack multiple graph convolution operations for multi-hop reasoning over the heterogeneous graph. To address the smoothing problem, we propose an enhanced gating mechanism that controls the connections between convolutional network layers. Finally, we enumerate possible entity combinations and incorporate a softmax classifier to get the relation of entity pairs. The contributions of this paper can be summarized as following:

- We propose a novel heterogeneous graph-based node-oriented model for CID relation extraction which simultaneous extract intra- and inter-sentence relation facts.
- We propose a gating mechanism for GCNs which can better capture the relation between long-distance nodes by alleviating the over-smoothing problem.
- We conduct wide experiments on a public document-level biomedical datasets. Experimental results show that the proposed method outperforms several strong baselines.

## 2. Related Work

Relation extraction is the widely studied task of automatically retrieving structured information (relational facts) from text. It has received widespread attention as the key component for building Knowledge Graphs. According to the text of input, relation extraction falls into sentence-level and document-level methods. The CID relation extraction is a recently introduced task. From the task definition (Wei et al., 2015), CID relations are typically determined at document level, meaning that this task should consider both intra- and inter-sentence relations.

Early studies tackle the CID relation extraction based on traditional sentence-level relation extraction methods. It is worth noting that for inter-sentence relations, multiple sentences are generally taken as a whole, and the cross-sentence features are extracted. The task is usually considered as a classification problem. Jiang et al. (2015) exploit word embeddings and linguistic features to represent the relation between chemicals and diseases. Then, they use a logistic regression model with a heuristic post-processing method to get the CID relations. Zhou et al. (2015) apply the shortest dependency path tree kernel with support vector machine (SVM) for the CID relation classification. Qian and Zhou (2016) incorporating different maximum entropy (ME) classifiers with lexical and syntactic features to extract cross-sentence relations. In addition, methods using prior knowledge and external resource have been proved to be effective. Pons et al. (2016) add prior knowledge about chemicals and diseases from a graph database. Peng et al. (2016) incorporate weakly labeled data to improve the performance. With the development of deep learning, (Zhou et al., 2016) propose a hybrid method which adopts LSTM to generate semantic representations. Gu et al. (2017) employ CNN to learn the context and dependency representations. Nguyen and Verspoor (2018) further use character-based word embeddings to improve the CNN model.

The above methods, especially the introduction of deep neural networks, have greatly promoted the development of this task. However, these methods ignore the interactions in multiple mentions of the target entities in different sentences. Recently, the graph-based approaches are proposed for the CID relation extraction. Quirk and Poon (2017) build a document graph with different dependency edges. They incorporate both standard dependencies and discourse relations and provide a unifying way to model relations intra- and inter-sentences. Peng et al. (2017) exploit graph LSTM to extract n-ary relations that span multiple sentences. Following this work, graph state LSTM (Song et al., 2018) and RNNs on dependency tree structures (Gupta et al., 2019) are used to model inter-sentence relations. These methods can simultaneously capture intra- and inter-sentential features, but they do not aggregate the features of multiple mentions. To address this shortcoming, Verga et al. (2018) form pairwise predictions over multiple sentences using a self-attention encoder, and aggregate the predictions by multi-instance learning. Christopoulou et al. (2019a) use an edge-oriented graph neural model to learn the representation of mention pairs. Nan et al. (2020) develop a refinement strategy to automatically induce the latent document-level graph, which helps to reason relations across sentences.

## 3. Task Definition

**Figure 1:**
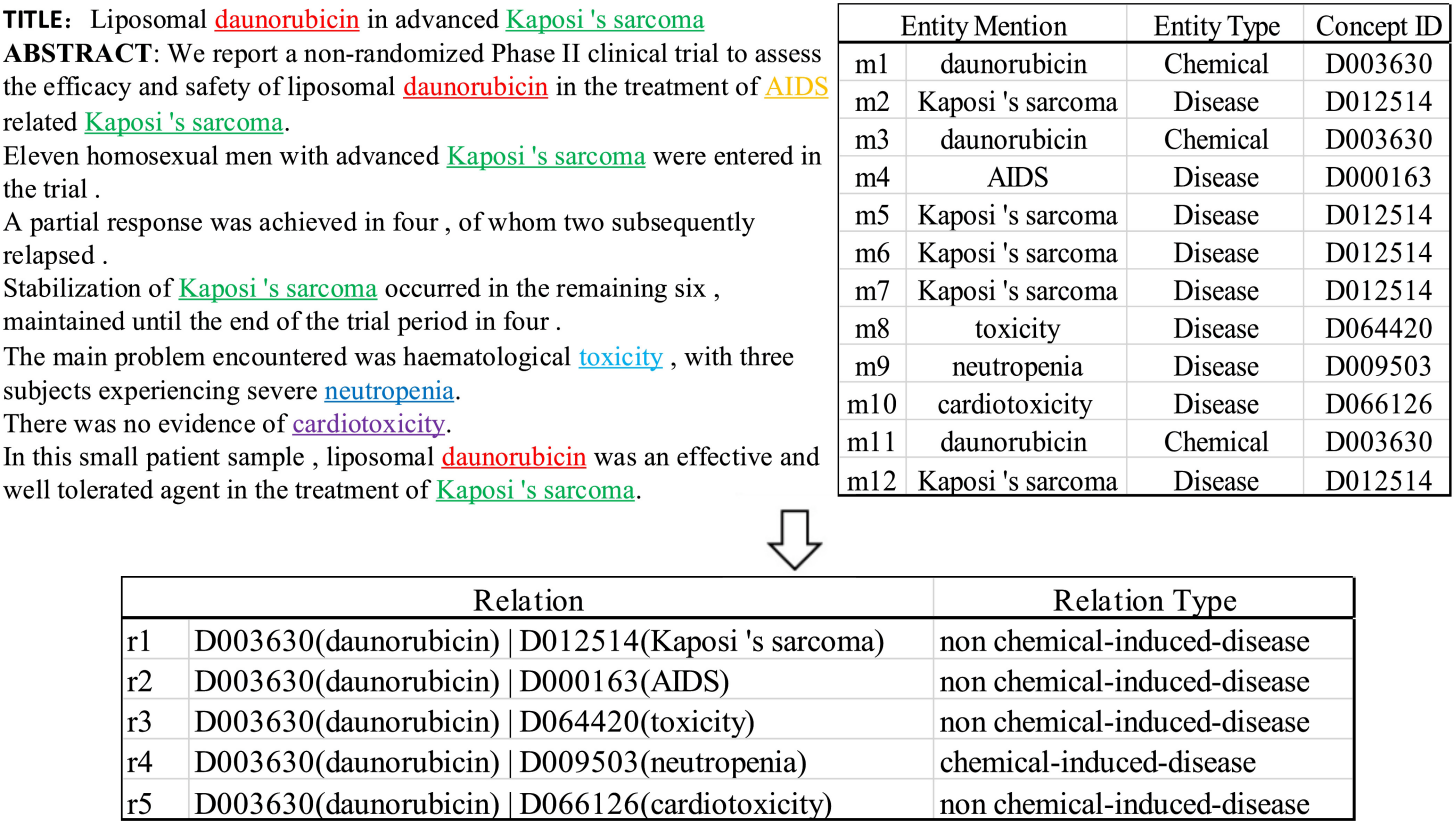
Introduction to document-level chemical-induced disease relation extraction.

We follow the definition of CID relation extraction from BioCreative V community. The input of CID relation extraction task is a well-annotated biomedical document from PubMed articles. The output is a ranked list < *chemical, disease*> pairs with normalized concept identifiers for which chemical-induced diseases are associated in the document. We introduce a document instance from CDR dataset and shown in Figure 1 as an example to help understand the task. As illustrated in the Figure 1, given a biomedical document composed with eight sentences and 12 chemical or disease entity mentions corresponding to six concept ID. In the document, entities may have multiple mentions scattered in different sentences with same color. The goal of CID task is to find CID relations in concept pairs (e.g., D003630 and D009503), rather than two mentions. In order to identify the relational fact < *D003630*; *chemical_induced_disease*; *D009503*>, *D003630* is chemical entity concept means daunorubicin and *D009503* is disease entity concept means neutropenia, one has to first identify the fact that *daunorubicin in advanced Kaposi 's sarcoma* is located in the title, then identify the facts *neutropenia* is the symptom of three subjects in the clinical trial from Sentence 5 in the abstract, and finally infer from these facts that *D003630* can induce *D009503*. Clearly, the process requires reading and reasoning over multiple sentences in the document.

Formally, the document-level chemical-induced disease (CID) relation extraction task can be formulated as follows. Given an input document *d* composed of *T* sentences *s*_1_, *s*_2_, ⋯ , *s*_*T*_, with *N* entity mentions *m*_1_, *m*_2_, ⋯ , *m*_*N*_, and *R* normalized entity concept identifiers *c*_1_, *c*_2_, ⋯ , *c*_*R*_. The task aims at extracting the relation *r* between each pair of chemical entity *c*_*i*_ and disease entity *c*_*j*_, for *i, j* = 1, 2, ⋯ , *R*. the relation *r* = 1 denotes that the chemical entity *c*_*i*_ and the disease entity *c*_*j*_ has the *Chemical-induced Disease* relation, *r* = 0 denotes there is no relation between two entities.

## 4. The Overview of Our Model

**Figure 2:**
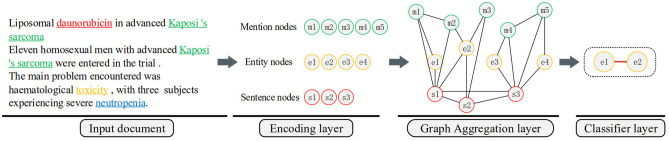
The overall architecture of the proposed neural model.

In this paper, we present an effective graph convolutional neural network for document-level chemical-induced disease relation extraction: CID-GCN. It consists of three modules, namely (i) encoding layer, (ii) graph aggregation layer, and (iii) classifier layer. Figure 2 illustrates the detailed structure of the model.

The purpose of the encoding layer is to learn the feature vector representation of the three nodes from the input document. The backbone of the nodes encoder network is an RNN. RNN is widely used for learning sequential and time-dependent structures inherent in the text, and achieved state-of-the-art results in many Natural language processing (NLP) tasks of high-value biomedical domain in recent years, including biomedical Named Entity Recognition (NER), biomedical QA etc. In order to construct a document-level graph, we encode three different types of nodes, respectively mention nodes, entity nodes, and sentence nodes. Specifically, given a document as the model input, it first generates a deep contextualized representation for each sentence using RNN with LSTM cell. Next, it constructs the representation of nodes based on the sentence representations. The specific details are explained in section 5.

The graph aggregation layer is devised to inference entity nodes interactions in the document. First, we construct a graph by connecting the graph nodes based on the natural associations among the three nodes in the document. After building the heterogeneous document-level graph, we utilize GCN to encode entity nodes by exploiting other interactions in the document. Recently, it has demonstrated that GCNs is powerful in processing relational reasoning on graphs. We stack multiple graph convolutional operations over the graph to map the node vectors into a set of new node representations. We will provide a brief recap of GCN and introduce its application in section 6.

Last, the output classifier module gives the relation prediction from two graph representations of entity nodes. In section 7, the loss function and the training process will be described further.

## 5. Encoding Layer

As we mentioned in section 4, encoding layer aims to learn the features related to specific three nodes.

### 5.1. Word Embeddings

Word embeddings are learned from a large amount of unlabeled data and have been shown to be able to capture the meaningful semantic regularities of words (Bengio et al., 2003; Erhan et al., 2010). The input tokens of the neural network model are a sequence of discrete variables. We usually transform these discrete variables into vector representations in the NLP area.

In this work, given a document *d*, we use NLTK python library^1^ to convert the corresponding multiple sentences *s*_1_, *s*_2_, ⋯ , *s*_*T*_ into multiple sequences of token, respectively. Then, we use the pre-trained word embeddings trained by two corpora: PubMed Central Open Access subset (PMC) and PubMed (Chiu et al., 2016). All the input word tokens will be transformed into low-dimensional vectors by looking up word embeddings tables, respectively, in sentence units. In this work, we denote the dimension of word embeddings by *d*_*w*_. These word embeddings of each sentence are transformed for the subsequent layers.

### 5.2. LSTM Encoding Layer

RNN has been widely exploited to deal with variable-length sequence input and successfully applied in various NLP tasks. The long-distance history is stored in a recurrent hidden vector which is dependent on the immediate previous hidden vector. LSTM (Hochreiter and Schmidhuber, 1997) is one of the popular variations of RNN to mitigate the gradient vanish problem of RNN. LSTM has three gates(input *i*, forget *f* and output *o*), and a cell memory vector *c*. The input gate can determine how incoming vectors *x*(*t*) alter the state of the memory cell. The output gate can allow the memory cell to have an effect on the outputs. Finally, the forget gate allows the cell to remember or forget its previous state. Given an input sequence **x** = {**x**_1_, **x**_2_, ⋯ , **x**_*n*_}, where **x**_1_ is a *d*_*w*_ dimension vector. The hidden vector **h**_*t*_ (the dimension is *d*_*h*_) at the time step *t*(1 ≤ *t* ≤ *n*) is calculated as follows:

(1) $$\begin{array}{l} \;i_{t}=\;\sigma (W_{i}x_{t}+U_{i}h_{t-1}+b_{i})\\ \;f_{t}=\sigma (W_{f}x_{t}+U_{f}h_{t-1}+b_{f})\\ \;o_{t}=\sigma (W_{o}x_{t}+U_{o}h_{t-1}+b_{o})\\ {\tilde{C}}_{t}=\;tanh(W_{c}x_{t}+U_{c}h_{t-1}+b_{c})\\ C_{t}=i_{t}⊙{\tilde{C}}_{t}+f_{t}⊙C_{t-1}\\ \;h_{t}=\;o_{t}⊙tanh(C_{t}) \end{array}$$

where $W_{i},W_{f},W_{o},W_{c}\in {\mathbb{R}}^{d\times h}$ and $U_{i},U_{f},U_{o},U_{c}\in {\mathbb{R}}^{h\times h}$ are weight parameters and $b_{i},b_{f},b_{o},b_{c}\in {\mathbb{R}}^{h}$ are bias parameters, and ⊙ denotes element-wise multiplication. The subscripts *i, f, o* represent input gate, forget gate and output gate, respectively.

In this work, given that a document contains *T* sentences *s*_1_, *s*_2_, ⋯ , *s*_*T*_, we do the following operations for each sentence. First, we use Bidirectional LSTM (BiLSTM) as the sentence encoder to read the input sequence in both left-to-right and reverse order. Second, we combine bidirectional information for each word by averaging the forward and the backward output. Thus, given a sentence ***s*_*i*_** = {*x*_1_, *x*_2_, ⋯ , *x*_*n*_} as a sequence of tokens, the LSTM encoding layer is responsible to map each token to the continuous embedding representations as **H** = {**h**_1_, **h**_2_, ⋯ , **h**_*n*_}. After encoding contextualized representations of all the sentences, There are three types of node that need to be constructed in CID-GCN.

#### 5.2.1. Mention Node Representation

The mention node is intended to represent different mentions of entities that appear in the document. A mention node is represented by the average of the hidden vectors of all words contained in the mention after LSTM encoding. Assuming that a document has *N* mentions, the representation of mention nodes is formed as *N*_*m*_*j*__ = [**avg**_*h*_*i*_∈*m*_*j*__(*h*_*i*_); *t*_*m*_]*j* = 1, 2, ⋯ , *N*. where *t*_*m*_ is a node type embedding for mention.

#### 5.2.2. Entity Node Representation

Similar to the structure of the mention node, the structure of the entity node is represented by the average of the representations of all the mentioned nodes corresponding to the entity. Assuming that a document has *R* entities, the representation of entity nodes is formed as *N*_*e*_*j*__ = [**avg**_*m*_*i*_∈*e*_*j*__(*m*_*i*_); *t*_*e*_]*j* = 1, 2, ⋯ , *R*. where *t*_*e*_ is an node type embedding for entity.

#### 5.2.3. Sentence Node Representation

A sentence node *n*_*s*_*j*__ is represented by the average of output at all times in **H_j_**: Assuming that a document has *T* sentences, the representation of sentence nodes is formed as *N*_*s*_*j*__ = [**avg**_*h*_*i*_∈*s*_*j*__(*h*_*i*_); *t*_*s*_]*j* = 1, 2, ⋯ , *T*. where *t*_*e*_ is an node type embedding for sentence.

## 6. Graph Aggregation layer

GCN is a powerful approach for mining the structural features on the graph. We use the GCN to capture the correlations of multiple entity nodes. In this section, we will introduce the structure of document graph and describe the preliminary and detail of the GCN.

### 6.1. Document Graph Construction

A graph is made up of nodes (also called vertices) which are connected by edges (also called links). Normally, it is an ordered pair G=(V,E), where V is a set of nodes and E is a set of edges. We can mathematically represent a graph with *n* nodes by an adjacency matrix **A** ∈ ℝ^*n*×*n*^, where **A**_*ij*_ = 1 if an edge exists between node *i* and *j*, otherwise 0.

In this paper, we construct a document-level heterogeneous graph by connecting the three nodes constructed in the section above. Specifically, we first constructed a total of *N* + *R* + *T* nodes of mention, entity, and sentence. Next, we connect the graph nodes based on the natural associations between the three nodes in the document to obtain the adjacency matrix **A** ∈ ℝ^(*N*+*R*+*T*) × (*N*+*R*+*T*)^ of the document graph. These natural connections have the following 5 situations:

- Mention-to-Mention. If there are two mentions appear in the same sentence, there is an implicit meaning that cannot be ignored. These pair of two mention nodes should be connected.
- Sentence-to-Sentence. We connect every sentence nodes to model global information.
- Mention-to-Sentence. Since every mention must appear in a sentence, we connect all mentions to the sentence node where they are located.
- Mention-to-Entity. Similar to Mention-to-Sentence, we connect all the mentioned nodes to their corresponding entity node.
- Entity-to-Sentence. To model the diversity of entities in the document, we connect all entities to the sentence node where their mentions have appeared.

### 6.2. Graph Convolutional Networks

GCN is an extension of convolutional neural network and can be operated to encode graphs. It carry out convolution filtering on the graph and update the node representations by propagating information between nodes. We stack all the three types of constructed nodes into the node set V. The set of nodes V are represented as *d* dimensional vectors **V** ∈ ℝ^(*N*+*R*+*T*) × *d*^, the GCN layer on a graph can be written as a non-linear function *f*(**V**, **A**). When considering the stacking of multiple GCN layer and after exploiting the convolutional operation proposed in Kipf and Welling (2017), the GCN can be represented as follows:

(2) $$\begin{array}{r} {\text{V}}^{l+1}=f\left({\text{V}}^{l},\text{A}\right)=\delta \left(\text{A}{\text{V}}^{l}{\text{W}}^{l}\right) \end{array}$$

where δ(·) denotes an activation function, which is chosen as LeakyReLU in our experiments. As a general rule, the superscript *l* indicates the layer number. **W**^*l*^ ∈ ℝ^*d*×*d*^ is the learnable parameters of the convolutional filter. Different graph convolution layers have different convolutional filters, which are numbered using superscript *l*. It is not difficult to see that multiplying the adjacency matrix is equivalent to adding the feature of its neighbor node to each node. For instance, if entity node 5 has two adjacent nodes: sentence node 1 and mention node 4, the Equation (2) can be represented in another way as:

(3) $$\begin{array}{r} V_{5}^{l+1}=\delta \left(a_{35}v_{3}^{l}{\text{W}}^{l}+a_{45}V_{4}^{l}{\text{W}}^{l}+a_{55}V_{5}^{l}{\text{W}}^{l}\right) \end{array}$$

where *a*_*ij*_ is the element at row *i* and column *j* of the adjacent matrix **A**, $V_{i}^{l}$ denotes the node representation corresponding to the *i*-th node of the *l*-th layer. In this case, the GCN aggregates all adjacent node information with the same convolution weights, and after that, the result is passed through one activation function to yield the updated node feature. In this way, the adjacent nodes in the graph affect each other, and the relation among entity nodes is learned after multiple layers of convolution operations.

Facts (Kipf and Welling, 2017; Li et al., 2018) have proved that the graph convolution is a special form of Laplacian smoothing, which mixes the features of the nodes and its neighbors.The smoothing operation makes the features of the nodes in the same cluster similar, thereby optimizing the classification task, which is the key reason why GCNs works so well. However, this also brings the potential problem of over-smoothing when stacking multiple GCN layers. The over-smoothing problems can lead to similar node representations, thus losing the discrimination of the node in the classification function. Moreover, this problem also limits the long-distance relation modeling ability of the model. However, long-distance reasoning paths are very common in the document graph we construct, because the relation between entity nodes may need to be inferred from multiple mention nodes and sentence nodes.

In order to alleviate the above problems, we propose a gating mechanism for GCNs. This mechanism divides the traditional graph convolutional layer into two steps. The first step, using the structure information of the graph to aggregate the information of adjacent nodes to passing messages on the graph, which is consistent with the operation of the traditional GCNs. The second step, using a gating mechanism to control the updating of node representations. The gating mechanism can be calculated as follows:

(4) $$\begin{array}{l} g_{l}=sigmoid(W_{g}V_{l+1}+U_{g}V_{l}+b_{g})\\ \;V_{l+1}=V_{l+1}⊙g_{l}+V_{l}⊙(1-g_{l}) \end{array}$$

where ${\text{W}}_{g}\in {\mathbb{R}}^{d\times d}$ and ${\text{U}}_{g}\in {\mathbb{R}}^{d\times d}$ are two learnable parameters. The gate **g**_*l*_ controls the new node representation **V**_*l*+1_ update of each layer by considering the node representations generated by the previous layer **V**_*l*_ and the current graph convolutional layer **V**_*l*+1_. The gating mechanism aims to save the distinguished local information belonging to the current node representations itself after each graph convolution operation. Combined with effective global information and unique local information, the model can better understand the document graph and learn more distinguishable node representations, thereby alleviating the over-smoothing problem caused by multi-layer GCNs. Besides, compared with the edge update mechanism of walk-base method (Christopoulou et al., 2019b), our proposed gating mechanism does not require manual tuning of hyperparameters to determine the contribution of each hop.

## 7. Classifier Layer

After multiple times of aggregation, we obtain a set of new representations of all the nodes. For each entity chemical-disease pair (ei,ej), we use a bilinear function to compute the probability for chemical-induced disease relation as:

(5) $$P(r|e_{i},e_{j})=softmax({e_{i}}^{T}W_{cls}e_{j})$$

where ${\text{W}}_{cls}\in {\mathbb{R}}^{d\times k\times d}$ is a learnable parameter matrix. *k* is the number of labels, which is 2 in this work cause CID relation extraction is a binary classification problem.

In this paper, we use the stochastic gradient descent (SGD) algorithm to minimize the log likelihood function. The loss function of our model is:

(6) $$\begin{array}{r} L=-\underset{d\in T}{\sum }\log p\left(r_{e_{i},e_{j}}=r_{e_{i},e_{j}}^{*}|d\right) \end{array}$$

where T represents the training set, $r_{e_{i},e_{j}}^{*}$ is the gold label for the relation between entity chemical-disease pair (ei,ej) in document *d*. During training, we minimize the loss function L of the gold CID relations.

## 8. Experiments

### 8.1. Datasets and Setting

We evaluate the performance of our model on **CDR** dataset proposed by BioCreative V. This dataset contains a total of 1,500 PubMed articles, 500 articles each for the training, development and test set. Each articles is manually annotated chemical mentions and disease mentions, the MeSH identifiers of chemical entity and disease entity, and the CID relation between the chemical entities and disease entities. Table 1 details the diseases and related annotations of these three data sets.

**Table 1:** Main results on CDR datasets.

**Model**	**Description**	**Precision**	**Recall**	**F1**
Zhou et al. (2015)	*CNN*	41.1	55.3	47.2
Zhou et al. (2016)	*LSTM+SVM*	64.9	49.3	56.0
Gu et al. (2017)	*CNN+Inter_ME+PP*	55.7	68.1	61.3
Nguyen and Verspoor (2018)	*CNN+Char*	57.0	68.6	62.3
Sahu et al. (2019)	*GCNN*	52.8	66.0	58.6
Peng et al. (2017)	*Graph LSTM*	62.1	64.2	63.1
Verga et al. (2018)	*BRAN*	55.6	70.8	62.1
Wang et al. (2020)	*GCN+Multihead Attention*	56.3	**72.7**	63.5
Christopoulou et al. (2019a)	*EoG*	62.1	65.2	63.6
Nan et al. (2020)	*LSR*	-	-	64.8
Our model	*CID-GCN*	**64.2**	66.4	**65.3**

*Bold marks highest number among all models*.

In our experiments, all hyper-parameters are tuned through cross validation on training set and development set. We initialize network with pre-trained embedding with a dimension of 300.The hidden state of one-side LSTM is 300. The sizes of the three types of node embedding is 50.The number of layers of the GCNs is 4. The experiments are trained with an NVIDIA RTX 2080Ti GPU. It took about 10 min per epoch.

### 8.2. Baselines and Evaluation Metrics

To evaluate the performance of the proposed method, we compare our model with six competitive baselines, as follows: (1) **CNN** (Zhou et al., 2015). (2) **LSTM+SVM** (Zhou et al., 2016). (3) **CNN+Inter_ME+PP** (Gu et al., 2017). (4) **CNN+Char** (Nguyen and Verspoor, 2018). (5) **GCNN** (Sahu et al., 2019). (6) **Graph LSTM** (Peng et al., 2017). (7) **BRAN** (Verga et al., 2018). (8) **GCN+Multihead Attention** (Wang et al., 2020). (9) **EoG** (Christopoulou et al., 2019a). (10) **LSR** (Nan et al., 2020). We use precision, recall, and F1 score to evaluate the performance.

### 8.3. Main Results

To evaluate the performance of the proposed method, we first compare our model with the baseline methods. The results are shown in Table 1, from which we can observe that:

(1) Compared with the current graph-based model, our model has achieved the best results. In detail, compared with *Graph LSTM* and *BRAN*, the improvements of our model are 2.2 and 3.2% in F1, respectively. It indicates that our method can better take advantage of the rich correlations among entities at document level. Furthermore, compared with *GCNN* and *GCN+Multihead attention* which both use plain-GCN, the improvements of our model are 6.7 and 1.8% in F1 score, respectively. This is due to our reasonable method for document graph construction. Similar to *EoG*, we construct a heterogeneous graph which contains mention, sentence and entity nodes. However, our model outperforms *EoG* with F1 score of 1.7%, and 2.1% in precision 1.2% in recall. The main reason is that the graph aggregation layer of our model can encode more entity-relation information for relation classification. The gating mechanism proposed in our model enables the aggregation layer to encode the complete graph structure without losing the information when modeling the information of multi-hop nodes. To further compare EoG with CID-GCN, we analyzed the performance of EoG and CID-GCN using different word embeddings. As shown in Table 2, CID-GCN is superior to EoG in random initialization *(random)*, general domain *(GloVe)* (Pennington et al., 2014), domain-specific *(PubMed)* (Chiu et al., 2016) word embeddings. *LSR* also exploits graph convolution operation on the document graph but uses the dense connection to address the over-smoothing problem, our model outperforms it with F1 score of 0.5%. Thus, Our method can make better capture the correlation between chemical entities and disease entities in the CDR dataset.

**Table 2:** Performance of EoG and CID-GCN with different pre-trained word embeddings.

**Model**	**F1 (%)**
EoG *(random)*	61.41
EoG *(GloVe)*	63.01
EoG *(PubMed)*	63.62
CID-GCN *(random)*	62.55
CID-GCN *(GloVe)*	64.46
CID-GCN *(PubMed)*	**65.32**

*Bold marks the highest number among all models*.

(2) Compared with the current non-graph-based model, our model also has achieved the best performance in CID relation extraction task. In fact, these methods do not perform as well as graph-based methods on the CDR dataset. Specifically, *CNN* is characterized by a low precision, which is caused by its deficiency in cross-sentence relation facts. The other three methods utilize different inter- and intra-sentence models and merge the final predictions. In the detailed analysis of the datasets, we find that 30% CID relations in the test set belong to entity pairs that cross sentences. Compared with *LSTM+SVM*, the improvements of our model is 9.3% in F1. In contrast, the performance gap of other baselines is relatively small, 4.0% and 3.0% in F1, respectively.

(3) In order to verify the superiority of our model in extracting cross-sentence CID relations, we separately verified the performance of the model in inter- and intra-sentence. We selected two comparison models from graph-based and non-graph-based models, *CNN+Inter_ME+PP* and *EoG*, respectively. Table 3 depicts the results of our proposed model, in comparison with the two baseline selected above. As it can be observed, *CNN+Inter_ME+PP* obtained a very low F1 score when recognizing the inter-sentence CID relation. The reason is it cannot well capture the interactions in multiple mentions of the target entities in different sentences. Compared with *EoG* and *BRAN*, the improvements of our model is also considerable.

**Table 3:** Experimental results in intra- and inter-sentence CID relations.

**Model**	**Intra (%)**				**Inter (%)**		
	**Precision**	**Recall**	**F1**	**Precision**	**Recall**	**F1**
CNN+Inter_ME+PP	59.7	55.0	57.2	51.9	7.0	11.7
EoG	64.0	73.0	68.2	56.0	**46.7**	50.9
Our model	**68.1**	**75.9**	**71.8**	**62.2**	45.2	**52.4**

*Bold marks the highest number among all models*.

Our model not only can simultaneous extract intra- and inter-sentence relation facts, but also capture better the interactions between entities regardless of whether they cross sentences. We further verify the advantages of the graph aggregation layer with different connect mechanism for GCNs in the following subsection. Therefore, our model outperforms all the baselines and more suited to the CID relation extraction task.

### 8.4. Ablation Experiments

To investigate the effectiveness of the graph aggregation layer proposed in CID-GCN, we conduct an ablation study using the test set of CDR dataset. Table 4 shows the results of ablation study. From the table, we find that:

**Table 4:** Ablation study for the graph aggregation layer, where “w/o” indicates without and “w/” indicates with.

**Model**	**Precision**	**Recall**	**F1**	**Intra-F1**	**Inter-F1**
Our model	**64.2**	**66.4**	**65.3**	**71.8**	**52.4**
w fully-connected	60.6	58.8	59.7	68.5	43.8
w/o gating mechanism	61.2	52.3	56.4	62.9	44.6
w/o aggregation layer	53.0	51.0	53.1	60.8	30.6

*Bold marks the highest number among all models*.

(1) When we change the document graph to a fully connected graph, even with the existence of the gating mechanism, the performance of the model still drops rapidly. This results show the importance of a reasonable document graph structure.

(2) Both intra-F1 and inter-F1 scores drop when we remove the gating mechanism. This results confirm the existence of the over-smoothing problem, and also show that the gating mechanism does enable the model to better capture the relation between long-distance nodes.

(3) Finally, when we remove the aggregation layer, inter-F1 scores drops dramatically. According to our statistics on the CDR dataset, there are more than 54% entities have multiple mentions in different sentences. This results prove our proposed graph aggregation layer can effectively extract the inter-sentence CID relation facts by reading and reasoning over multiple sentences in the document graph we construct.

### 8.5. Analysis Using GCN

**Figure 3:**
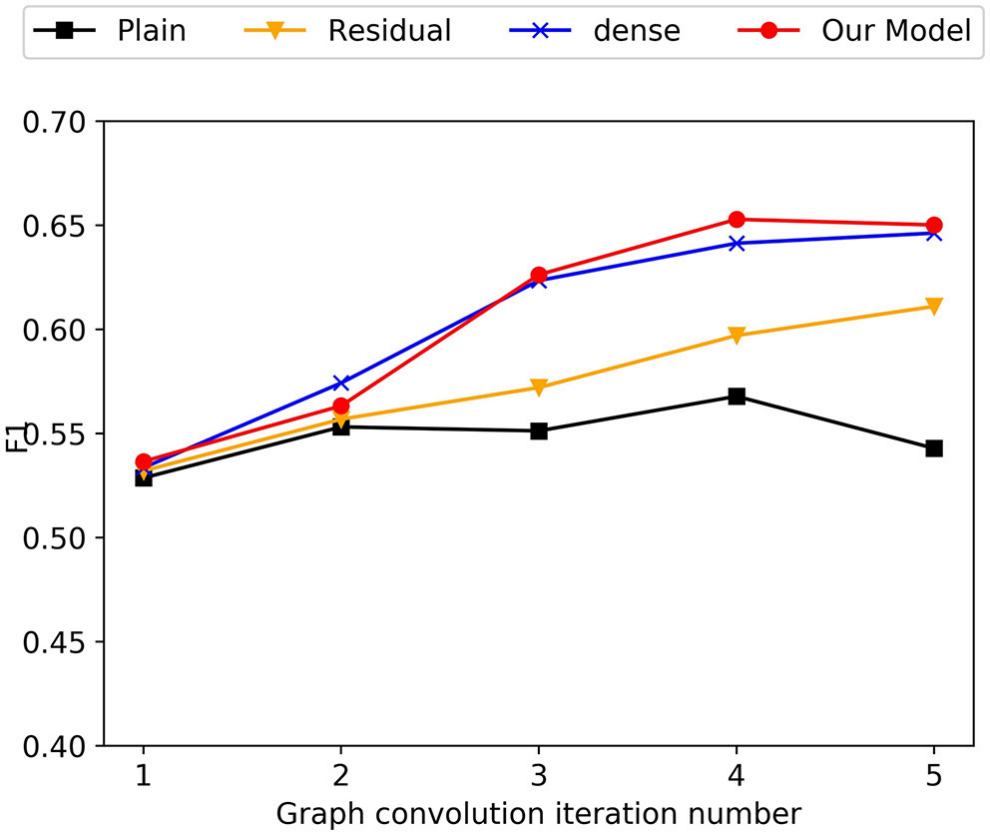
Experimental results of different GCN connection mechanisms.

Recently, GCNs have shown excellent performance in various NLP tasks. Though effective, most of the current GCN models are shallow due to the smoothing problem. As illustrated in the Figure 3, our model also suffers from over-smoothing problems when using plain-GCN. The plain-GCN achieve their best performance with 4-layer models, but the performance is still poor. In the heterogeneous graphs we construct, we give various nodes different meanings, which may aggravate the over-smoothing problem.

To alleviate this problem, we tried three different connection methods. (1) Similar to ResNet, we add residual connections between different layers of GCNs. (2) Similar to DenseNet, we concatenates the outputs of all graph convolution layers to get the final mention, sentence and entity node representations. (3) Inspired from the forget gate in LSTM, we propose a gating mechanism for GCNs.

We conduct experiments to investigate the effectiveness of the three enhanced connect mechanisms on different layers. As shown in the Figure 3, the three enhanced connect mechanisms slows down the over-smoothing problem to varying degrees. Among them, our proposed gating mechanism achieves the best performance on 4-layer GCNs. It means the gating mechanism we proposed is more suited to the CID relation extraction task and the document graph we construct.

### 8.6. Case Study

In order to investigate the advantages of our model, we conduct case study on the test set of CDR. Compared to all the baselines, our model can extract more inter-sentence CID relations correctly. For example, in the document number 57355 entitled “Long-term propranolol therapy in pregnancy : maternal and fetal outcome.” The main idea of this article is to study the relationship between 6 diseases and long-term propranolol treatment during pregnancy through two sets of experiments. There are 9 sentences in this document, and the first 8 sentences contain a mention of the same chemical entity “propranolol (D011433).” The experiment of this document uses exclusion division to exclude the first 5 diseases. The last sentence “Growth retardation, however, appears to be significant in both of our series” finally pointed out the CID relation between chemical entity “propranolol (D011433)” and disease entity “Growth retardation (D005317)” and it do not contain “propranolol (D011433)” entity. As can be seen from this case, our model can correctly extract the inter-sentence CID relations by reading and reasoning over multiple sentences in the document.

## 9. Conclusion

In this paper, we propose CID-GCN, an effective Graph Convolutional Networks with gating mechanism, for CID relation extraction. First, we construct a heterogeneous graph which contains mention, sentence and entity nodes and connect the nodes based on the natural associations among the three nodes in the document. In particular, in order to solve the over-smooth problem of graph convolutional neural networks on heterogeneous graphs, we propose a gating mechanism to connect different GCN layers. Experimental results on CDR datasets indicate that our proposed model is effective and outperforms several strong baseline methods.

## Data Availability Statement

Publicly available datasets were analyzed in this study. This data can be found here: http://ctdbase.org/.

## Author Contributions

DZ: conceptualization and methodology. CZ: writing the original draft and experiments. ZQ: investigation and visualization. All authors have read and approved the final manuscript.

## Conflict of Interest

The authors declare that the research was conducted in the absence of any commercial or financial relationships that could be construed as a potential conflict of interest.

## Footnotes

^1^https://www.nltk.org
